# EIF2A-dependent translational arrest protects leukemia cells from the energetic stress induced by NAMPT inhibition

**DOI:** 10.1186/s12885-015-1845-1

**Published:** 2015-11-05

**Authors:** Chiara Zucal, Vito G. D’Agostino, Antonio Casini, Barbara Mantelli, Natthakan Thongon, Debora Soncini, Irene Caffa, Michele Cea, Alberto Ballestrero, Alessandro Quattrone, Stefano Indraccolo, Alessio Nencioni, Alessandro Provenzani

**Affiliations:** 1 Laboratory of Genomic Screening, CIBIO, University of Trento, Trento, Italy; 2 Laboratory of Molecular Virology, CIBIO, University of Trento, Trento, Italy; 3 Department of Internal Medicine, University of Genoa, Genoa, Italy; 4 Laboratory of Translational Networks, CIBIO, University of Trento, Trento, Italy; 5 Istituto Oncologico Veneto IOV-IRCCS, Padova, Italy

**Keywords:** NAMPT, EIF2A, AMPK, Energetic stress, Translation arrest, UPR

## Abstract

**Background:**

Nicotinamide phosphoribosyltransferase (NAMPT), the rate-limiting enzyme in NAD^+^ biosynthesis from nicotinamide, is one of the major factors regulating cancer cells metabolism and is considered a promising target for treating cancer. The prototypical NAMPT inhibitor FK866 effectively lowers NAD^+^ levels in cancer cells, reducing the activity of NAD^+^-dependent enzymes, lowering intracellular ATP, and promoting cell death.

**Results:**

We show that FK866 induces a translational arrest in leukemia cells through inhibition of MTOR/4EBP1 signaling and of the initiation factors EIF4E and EIF2A. Specifically, treatment with FK866 is shown to induce 5′AMP-activated protein kinase (AMPK) activation, which, together with EIF2A phosphorylation, is responsible for the inhibition of protein synthesis. Notably, such an effect was also observed in patients’ derived primary leukemia cells including T-cell Acute Lymphoblastic Leukemia. Jurkat cells in which AMPK or LKB1 expression was silenced or in which a non-phosphorylatable EIF2A mutant was ectopically expressed showed enhanced sensitivity to the NAMPT inhibitor, confirming a key role for the LKB1-AMPK-EIF2A axis in cell fate determination in response to energetic stress *via* NAD^+^ depletion.

**Conclusions:**

We identified EIF2A phosphorylation as a novel early molecular event occurring in response to NAMPT inhibition and mediating protein synthesis arrest. In addition, our data suggest that tumors exhibiting an impaired LBK1- AMPK- EIF2A response may be especially susceptible to NAMPT inhibitors and thus become an elective indication for this type of agents.

**Electronic supplementary material:**

The online version of this article (doi:10.1186/s12885-015-1845-1) contains supplementary material, which is available to authorized users.

## Background

Aberrant activation of metabolic pathways has emerged as an hallmark of proliferating cancer cells and pharmaceutical approaches targeting cell metabolism hold potential for treating cancer [1]. Nicotinamide adenine dinucleotide (NAD^+^) plays a key role in different biochemical processes, acting as a coenzyme in redox reactions or as a substrate for NAD^+^ degrading enzymes, such as poly(ADP-ribose) polymerases (PARPs), cluster of differentiation 38 (CD38), and sirtuins. Intracellular NAD^+^ is continuously replenished utilizing either tryptophan, nicotinamide, nicotinic acid or nicotinamide riboside as a substrate [2], and nicotinamide phosphoribosyltransferase, NAMPT, is the rate-limiting enzyme for NAD^+^ biosynthesis from nicotinamide in mammalian cells [3]. High NAMPT levels, whose activity appears to be also important in the differentiation of myeloid cells [4], were shown to be required to support cancer cell growth, survival and epithelial-mesenchymal transition (EMT) transition [5, 6], and have been reported in different types of tumors [7, 8]. In line with these notions, several studies have highlighted a strong activity of NAMPT inhibitors in preclinical models of inflammatory and malignant disorders, including leukemia [2, 9–11]. FK866, a prototypical NAMPT inhibitor, was found to promote cell death in both lymphoid- and myeloid-derived hematological malignancies and its activity clearly resulted from intracellular NAD^+^ depletion [12–14]. Notably, opposite to cancer cells, activated immune cells [10], along with many other types of healthy cells, such as hematopoietic stem cells [12], appear unaffected by NAMPT inhibitors, and consistently, agents such as FK866 or CHS-828 are well tolerated in patients [15, 16].

The molecular consequences upon NAMPT inhibition are only partially understood. The induced NAD^+^ depletion clearly affects intracellular ATP levels resulting in mitochondrial dysfunction and activation of cell death pathways: reactive oxygen species generation and activation of the apoptotic cascade have both been involved in cell demise in response to NAMPT inhibitors [17]. ATP depletion has been related to the loss of plasma membrane homeostasis invariably leading to oncosis cell death [18]. Different groups have suggested a role for autophagic cell death in the cytotoxic activity of these drugs [10, 12, 13, 19]. In particular, Cea and colleagues proposed that FK866 would induce autophagy *via* activation of transcription factor EB (TFEB), a master regulator of the lysosomal-autophagic pathway [20], and through MTORC1/AKT and ERK1/2 pathway inhibition [21]. There is also evidence that AMP-activated protein kinase (AMPK), an important coordinator of metabolic pathways in response to energetic fluctuations [22], is activated by FK866 in prostate cancer cells affecting lipogenesis [23] and in hepatocarcinoma cells with impact on MTOR/4EBP1 signaling [24]. Moreover, NAMPT-dependent AMPK activation associated with deacetylation of liver kinase B1 (LKB1), an upstream kinase of AMPK, has been linked with modulation of NAD levels and with significant impact on neuron cell survival [25]. Translation inhibition is often observed during cell stress [26] and this event often involves a re-programming of translation leading to differential regulation of mRNAs, occurring also *via* alternative mechanisms, aimed at reorganizing cell physiology to respond to the insult.

In this study, we focused on the pre-toxic molecular events induced by FK866 in acute lymphoblastic leukemia cells, known to be sensitive to the drug [10], in order to define the molecular mechanism favoring cell death or cell survival. A marked global protein synthesis inhibition represented an early cellular response associated with the FK866-induced energetic stress and here we show that AMPK-EIF2A is a central hub in mediating this effect and is responsible for cell fate decisions.

## Methods

### Cell lines, primary B-CLL cell and T-ALL PDX isolation

Human Jurkat T-cell acute lymphoblastic leukemia (T-ALL) cells were purchased from the InterLab Cell Line Collection bank (ICLC HTL01002). SUP-T1 cells were purchased from ATCC (CRL-1942) and Molt-4 Clone 8 from NIH AIDS Reagent Program (Catalog #: 175). Human lung carcinoma A594 (CCL-185) and H460 (HTB-177) cells were purchased from ATCC. These cells were transduced with retroviral vectors encoding either LKB1 cDNA (pBABE-LKB1) or the pBABE control vector. Cell lines were grown in complete RPMI 1640 (Gibco Life Technologies) supplemented with 10 % fetal bovine serum (FBS, Lonza), 2 mM L-glutamine, 100 U/ml penicillin-streptomycin (Lonza). All cell lines were grown at 37 °C under 5 % CO_2_ and regularly tested for mycoplasma contamination. For primary B-CLL cell isolation, a 5 ml blood sample was obtained from patients presenting with marked lymphocytosis (>20000/μl) according to a protocol that was approved by the Ethics Committee of the Hospital IRCCS AOU San Martino IST in Genoa (#840, February 18th 2011). Patients’ written informed consent was collected. B-CLL cells were isolated by density gradient centrifugation on Ficoll-Hypaque (Biotest). The phenotype of the obtained cell preparations was confirmed by immunostaining with anti-CD19, anti-CD5, and anti-CD23 (Immunotech), and subsequent flow cytometric analysis. T-ALL xenografts (PD T-ALL) were established from BM (bone marrow) of newly diagnosed ALL pediatric patients, according to a protocol approved by the ethics committee of the University of Padova (Project number 16B/2013). The PD T-ALL cells used in this study have been published elsewhere [27]. At time of PD T-ALL establishment, written informed consent was obtained from the parents of the children. *In vitro* studies were performed with T-ALL cultures established from the spleen of the xenografts. Purity of the cultures (in terms of percentage of human CD5+ cells) was checked by flow cytometry and was always >85 %. Research carried out on human material was in compliance with the Helsinki Declaration.

### Chemicals

FK866 (sc-205325) was bought from Santa Cruz, Compound C (P5499), Nicotinic acid (N0761), Actinomycin D (A9415), (S)-(+)-Camptothecin (C9911), Cycloheximide (C1988), MG-132 (M7449), Doxorubicin hydrochloride (D1515) and Dexamethasone (D4902) were bought from Sigma-Aldrich, CHS-828 (200484-11-3) from Cayman chemical, Torin 1 (S2827) and Rapamycin (S1039) from Selleck Chemicals, Cisplatin (ALX-400-040) from Enzo Life Sciences and Propidium Iodide Staining Solution from BD Pharmingen. Jurkat cells were treated with drugs dissolved in DMSO at the same cell density (5X10^5^ cells/ml).

### Viability assays

Cell viability was assessed with the Annexin V-FITC Apoptosis Detection Kit I and 7-Aminoactinomycin D (7-AAD) Staining Solution (BD Pharmingen) according to manufacturer’s instruction. EC_50_ values of FK866 were determined by nonlinear regression analysis (GraphPad Prism software v5.01,) *vs* viable cells in mock conditions (DMSO).

Jurkat, A549 and H460 cell lines were grown and treated in 96 well-plate for 48 h. Cells were then assayed for viability using Thiazolyl blue tetrazolium bromide (MTT) M5655 (Sigma). In brief, MTT (5 mg/ml) at 10 % volume of culture media was added to each well and cells were further incubated for 2 h at 37 °C. Then 100 μl of DMSO was used to dissolve formazan. Absorbance was then determined at 565 nm by microplate reader. Cell survival was calculated and EC_50_ values were determined.

### Determination of NAD^+^-NADH and ATP levels and caspase/protease activity

Intracellular NAD^+^-NADH content was assessed with a NAD^+^-NADH Quantification Kit (BioVision) according to the manufacturer’s protocol. Intracellular ATP content was determined using Cell titer Glo Luminescent Cell Viability Assay (Promega). NAD^+^-NADH and ATP values were normalized to the number of viable cells as determined using Trypan Blue (Lonza). EnzChek Protease Assay Kit, containing a casein derivative labeled with green-fluorescent BODIPY FL (Life Technologies), was used to determine protease activity after treatment of 2x10^6^ cells. Cells were washed once with PBS and lysed in 500 μl of 1X digestion buffer, sonicated and centrifuged for 5 min at maximum speed. One μl of the BODIPY casein 100X was added to 100 μl of the supernatant and incubated for 1 h protected from light. Fluorescence was measured and normalized to protein concentration in the cell lysates (Bradford Reagent, Sigma). Caspase-Glo 3/7 Assay (Promega) was used to quantify caspase activity.

### RNA and protein click-iT labeling kits

Click-iT RNA Alexa Fluor 488 Imaging Kit (Life Technologies) was used to quantify the level of global RNA synthesis by flow-cytometry. Jurkat cells (3x10^6^/sample) were treated for 45 h with FK866 (or DMSO) and then incubated for 3 h with 1X EU working solution without removing the drug-containing media. EU detection was performed following the manufacturer’s protocol after cell fixation and permeabilization. Click-iT AHA Alexa Fluor 488 Protein Synthesis Assay (Life Technologies) was used to measure the rate of translation. Cells (3x10^6^/sample) were treated for 45 h with FK866, centrifuged and incubated for 3 h with 50 μM AHA in L-methionine-free medium (RPMI Medium 1640, Sigma-Aldrich) containing the drug (or DMSO). After fixation and permeabilization, AHA incorporation was assessed by flow cytometry. 7-AAD Staining Solution (0.25 μg/sample) allowed the exclusion of non-viable cells.

### Western blotting, antibody list and plasmids

Cells were lysed for 5 min on ice in RIPA lysis buffer supplemented with Protease Inhibitor Cocktail (Sigma-Aldrich). After sonication and clarification, equal amounts of proteins were separated by SDS–PAGE and blotted onto PVDF membranes (Immobilon-P, Millipore), as in [28]. The antibodies used were: 4EBP1 (sc-6936), p-4EBP1 (Ser 65/Thr 70; sc-12884), EIF4E (sc-9976), p-EIF4E (Ser 209; sc-12885), AKT1/2/3 (sc-8312), p-AKT1/2/3 (Ser 473; sc-7985), MTOR (sc-8319), BCL-2 (sc-509), NAMPT (sc-130058) from Santa Cruz; EIF2S1 (ab26197), p-EIF2S1 (Ser 51; ab32157), and p-MTOR (Ser 2448; ab1093) from Abcam; AMPKα (2603), p-AMPKα (Thr 172; 2531), ACC (3676) and p-ACC (Ser 79; 3661) and MCL1 (4572) from Cell Signaling. A mouse anti-β-actin antibody (3700, Cell Signaling) was used as a protein loading control. eIF2a 1 (Addgene plasmid # 21807), eIF2a 2 (Addgene plasmid # 21808) and eIF2a 3 (Addgene plasmid # 21809) were a gift from David Ron. A549 cells were transfected using Lipofectamine 3000 Reagent from Life Technologies. Cells were plated in 6 well and transfected at 70 % confluence for 24 h with 1 μg of DNA. Jurkat cells were transfected for 48 h with 1 μg of DNA in 24- well plate.

### Real-time PCR

Total RNA was extracted with Quick-RNA MiniPrep kit (Zymo Research) and treated with DNAse. cDNA was synthesized using RevertAid First Strand cDNA Synthesis Kit (Fermentas) following the manufacturer’s recommendation. Real-time PCR reactions were performed using the KAPA SYBR FAST Universal qPCR Kit on a CFX96 Real-Time PCR Detection System (BioRad). Relative mRNA quantification was obtained with the ΔCq method using β-actin (*ACTB*) as housekeeping gene. Primers’ sequences are reported as follows: *BiP/Grp78* (Fw: TGTTCAACCAATTATCAGCAAACTC Rev: TTCTGCTGTATCCTCTTCACCAGT) *ACTB* (Fw: CTGGAACGGTGAAGGTGACA Rev: AGGGACTTCCTGTAACAATGCA) *STK11*/*LKB1* (Fw: GAGCTGATGTCGGTGGGTATG Rev: CACCTTGCCGTAAGAGCCT).

### Lentiviral particles production and luciferase assay

Lentiviral particles were produced using the pHR-SIN-*R*-Myc-F, pHR-SIN-F-HCV-*R* and pHR-SIN-F-CrPV-*R* transfer vectors [29], coding for reporter genes controlled by a cMyc-5′UTR, HCV or CrPV IRESes regulated translation, by co-transfection of 293 T cells with the packaging plasmid pCMV-deltaR8.91 and the VSV envelope-coding plasmid pMD2.G. Five thousand Jurkat cells/sample were transduced. After treatment with FK866, luciferase activity was measured using the Dual-Glo Luciferase Assay System (Promega) and normalized for protein concentration.

### Silencing with shRNAs

The pLKO.1-based lentiviral plasmids containing AMPKa1 shRNA (TRCN0000000859), AMPKa2 shRNA (TRCN0000002169) or NAMPT shRNA expression cassette (TRCN0000116180) and (TRCN0000116181) were purchased from Sigma-Aldrich. Scramble shRNA (Addgene plasmid **#**1864 [30]) was used as a control. Vectors were produced in 293 T cells by cotransfection of the different transfer vectors with the packaging plasmid pCMV-deltaR8.91 and the VSV envelope-coding plasmid pMD2.G. 1 million of Jurkat cells were transduced with lentiviral particles expressing the control (shSCR) or NAMPT-silencing short hairpin RNA (shNAMPT) by spinning them down with vector-containing supernatants for 2 h at 1600xg at room temperature and leaving them incubate overnight at 37 °C without replacing the transduction supernatant. After changing the medium, the cells were further incubated for 72 h before collection for WB.

For AMPK silencing experiments, Jurkat cells were first transduced with the shRNA vector targeting the α1 subunit (shAMPKα1) as reported before. After 24 h from the first transduction the cells were then transduced again, following the same protocol, with the lentiviral vector coding for the shRNA targeting the AMPK α2 subunit (shAMPKα2). After changing the medium the next morning, the cells were further incubated for 48 h and then treated for additional 48 h with or without (DMSO) 5 nM of FK866.

To obtain LKB1 silencing, pLKO.1 transfer vectors were prepared by cloning annealed oligos coding for shRNAs (clone TRCN0000000408 for LKB1-A and clone TRCN0000000409 for LKB1-B) into the TRC cloning vector (Addgene plasmid #10878 [31] according to the TRC standard protocol. Cells were transduced by spinning them down with vector-containing supernatants and leaving them incubate overnight. After changing the medium, the cells were incubated for 72 h and then treated for additional 48 h with or without FK866.

### Statistical analysis

Experiments were performed in biological triplicates. *T*-test was used to calculate final *p*-values, without assuming variances to be equal (Welch’s *t*-test). *P*-value <0.05 was considered statistically significant.

## Results

### Sensitivity of leukemia cells to the NAMPT inhibitor FK866

**Fig. 1:**
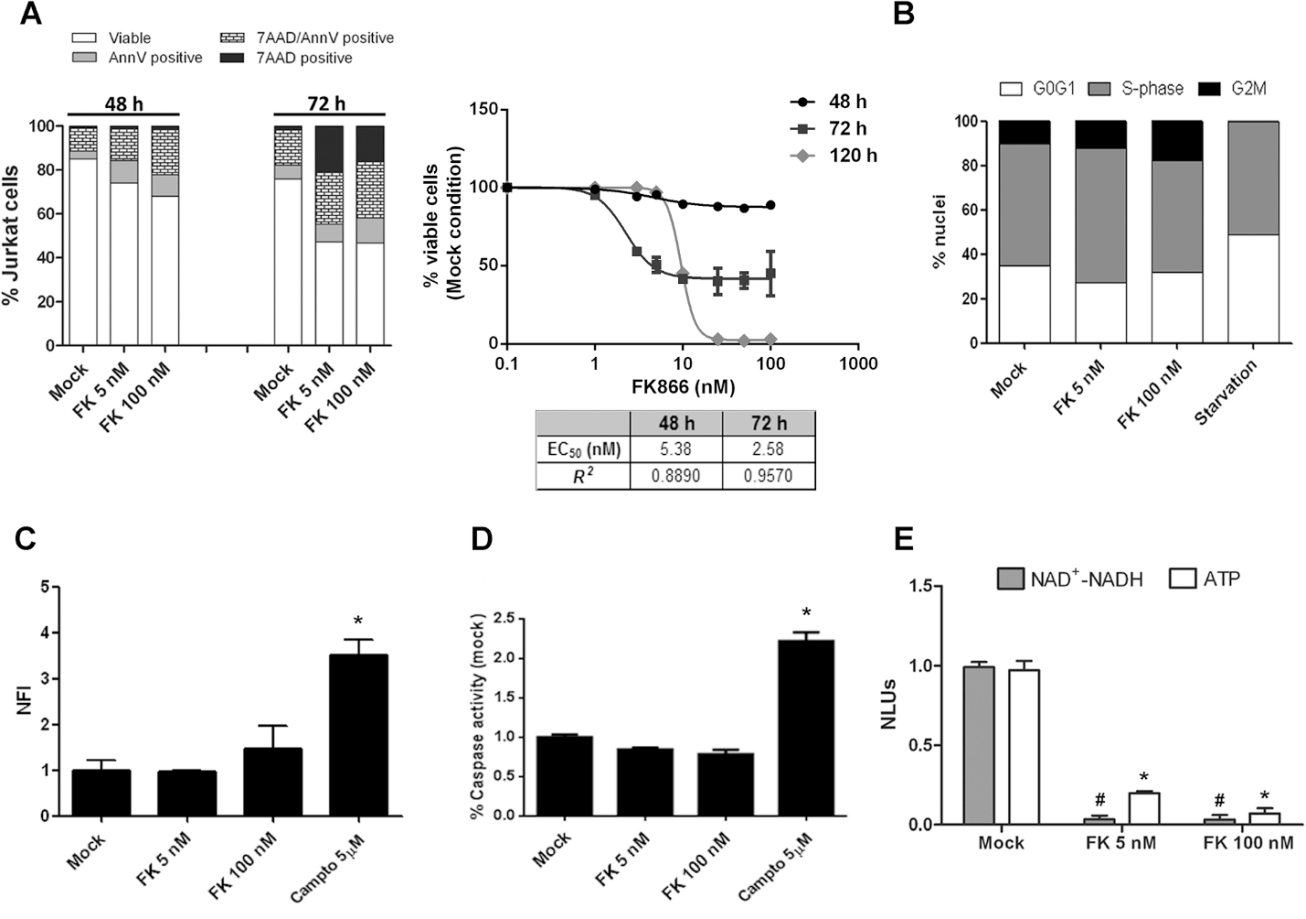
FK866 affects NAD^+^(H) and ATP levels in Jurkat cells leading to cell death. **a** Flow-cytometric quantification of cell viability with AnnexinV (FITC) and 7AAD (PerCP-Cy5-5-A) staining. Jurkat cells were treated with FK866 for 48,72 and 120 h. Mock, 5 nM FK866 and 100 nM FK866 (at 48 and 72 h) are shown as representative samples. Flow-cytometry experiments were carried out on two biological replicates and statistics were based on the acquisition of 10000 events/sample. **b** Cell-cycle analysis with PI staining of the nuclei after 48 h of treatment. Overnight serum starvation is shown as positive control of induced cell cycle synchronization in G0/G1 phase. Histograms quantify the cell cycle phase distribution. Flow-cytometry experiments were carried out on two biological replicates and statistics were based on acquisition of 30000 events/sample. Cell cycle phase analysis was done using ModFit LT 3.2 software and the Sync Wizard model. **c** Jurkat cells were treated with FK866 for 48 h. Alternatively, cells were exposed to 5 μM of Camptothecin for 4 h as a positive control. Protease activity in cell extracts was assessed with a commercially available kit and values were normalized to the protein concentration in the same extracts. **d** Caspase 3/7 activity was measured in Jurkat cells treated as in **c**. **e** Jurkat cells were treated with FK866 for 48 h. Thereafter, intracellular NAD^+^(H) and ATP levels were evaluated in comparison with control Jurkat cells. RLUs were normalized to number of viable cells. In **c**-**e** the means with SD of at least three independent experiments are shown. Statistical significance was calculated with *t*-test (* and # indicates *p*-value <0.05)

FK866 was previously shown to have cytotoxic activity at nanomolar concentrations against different types of hematological malignancies, including myeloid and lymphoid leukemias and multiple myeloma [12, 21]. We monitored FK866-induced cell death in Jurkat cells by quantifying early and late apoptosis with 7AAD and Annexin V staining. In line with previous reports, FK866 cytotoxic activity started to become evident between 48 and 72 h of exposure with approximately 74 and 47 % of viable cells left at these time points when cells are treated with FK866 100 nM (Fig. 1a), respectively. This suggests the existence of a lag phase through which cells can cope with the energetic shortage. Starting from a concentration of 10 nM, FK866 cytotoxic activity reached a plateau and an EC_50_ of 5.3 nM could be estimated after 48 h of exposure (Fig. 1a). Indeed, at 120 h we measured an effective IC_50_ of 10 nM, highlighting the inability of these cells to compensate for the energetic stress induced by FK866 in long term treatment (Fig. 1a). Cell cycle analysis of FK866-treated cells, at 48 h, showed a non-significant accumulation of cells in G2/M phase, while, as predicted, serum starvation resulted in accumulation of cells in G0/G1 phase (Fig. 1b and Additional file 1A). Forty-eight hours treatment with FK866 led to approximately 25 % of cell death, but did not lead to massive protease or caspase activation (Fig. 1c and d). However, 5 nM FK866 was sufficient to effectively reduce NAD^+^(H) and ATP levels in Jurkat cells, representing a pre-toxic experimental condition to apply for further experiments (Fig. 1e).

### FK866 and NAMPT ablation blocks cap-dependent translation, but not gene transcription, through MTOR/4EBP1, EIF4E, and EIF2A inhibition in cancer cells

**Fig. 2:**
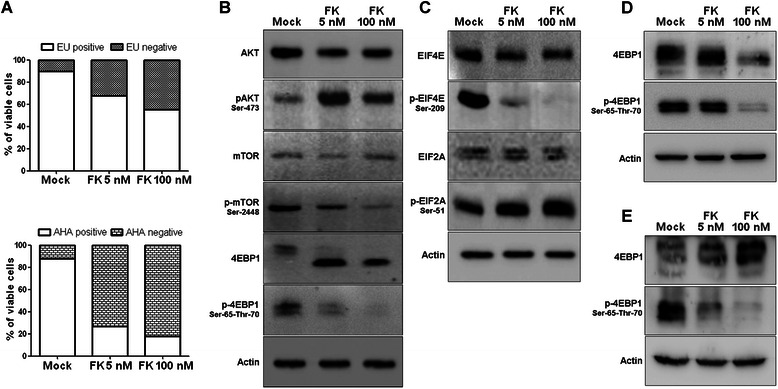
FK866 inhibits the signaling cascades controlling protein synthesis in T-ALL cell lines. **a** RNA synthesis was determined by monitoring EU incorporation with *Click-it* chemistry. Jurkat cells were treated for 48 h with or without (Mock) the indicated concentration of FK866 or for 3 h with 5 μM Actinomycin D, an RNA synthesis blocking agent. The histogram quantifies the dose-dependent transcription inhibition induced by FK866 in the viable cell population. In the lower part, *Click-it* chemistry based on the incorporation of an aminoacid analog (AHA) was used to monitor protein synthesis. Jurkat cells were treated for 48 h with or without (Mock) the indicated concentration of FK866 or for 3 h with 350 μM Cycloheximide, as a positive control for protein synthesis inhibition. The histogram quantifies FK866-induced protein synthesis arrest in the viable cell population. Flow-cytometry experiments were carried out on two biological replicates and statistics were based on acquisition of 50000 events/sample. **b** Jurkat cells were treated for 48 h with or without (Mock) the indicated concentration of FK866. Thereafter, cells were lysed and the levels of total and p-Akt (Ser-473), total and p-MTOR (Ser-2448), total and p-4EBP1 (Ser-65 and Thr-70), **c**) total and p-EIF4E (Ser-209), total and p-EIF2A (Ser-51) were detected by immunoblotting. **d** Molt-4 cells were treated with FK866 for 48 h and the levels of total 4EBP1 and p-4EBP1 were evaluated. **e** Western blot analysis as in **d** in SupT1 cells. **b**-**e**, one representative experiment out of at least three biological replicates is presented and β-actin was used as loading control

We assessed the impact of FK866 on global transcriptional and translational efficiencies in Jurkat cells. Global RNA transcription and translation were monitored using the *Click-it* chemistry and flow-cytometry by the incorporation of the nucleoside analog 5‑ethynyl uridine (EU) and of an aminoacid analog (AHA), respectively. In the viable Jurkat cell population, FK866 caused a reduction in the incorporation of EU in a dose–dependent manner, with 70 and 55 % of transcriptionally active cells in the presence of 5 and 100 nM FK866, respectively. Thus, despite NAD^+^ and ATP depletion, cells treated with FK866 for 48 h essentially retained their ability to perform RNA transcription. By contrast, even 5 nM FK866 determined a striking reduction (up to 30 %) of the fraction of viable cells showing active protein synthesis (Fig. 2a and Additional file 1B). The utilization of bicistronic reporter assays to test the efficacy of cap or IRES (Internal Ribosome Entry Site) dependent translation confirmed that FK866 induced a strong translation arrest with a major impact on cap-dependent translation in Jurkat cells (Additional file 2).

Since the initiation phase is considered the limiting step of translation [32], we evaluated the activation of three signaling pathways regulating the canonical cap-dependent translation process. The Mammalian Target of Rapamycin (MTOR) kinase regulates the p70 ribosomal S6 kinase (p70-S6K) and the eukaryotic translation initiation factor 4E-binding protein 4EBP1, whose phosphorylation determines EIF4E availability for its interacting partner EIF4G, which is involved in mRNA recruitment to the ribosomes for protein translation [32]. It has been recently shown that FK866 induces MTOR de-phosphorylation [24], thereby inducing autophagic cell death in multiple myeloma cells [21, 33]. As shown in Fig. 2b, Jurkat cells treated with FK866 indeed showed a marked de-phosphorylation of MTOR and 4EBP1. Enhanced AKT phosphorylation at Ser-473 was also observed (Fig. 2b), which is in line with the paradoxical activation of AKT by MTORC2 complex following inhibition of MTOR as reported with different MTOR inhibitors in multiple myeloma cells [34]. Notably, treatment with FK866 led to a previously unappreciated de-phosphorylation of EIF4E on serine 209, suggesting that the MAP Kinase Interacting Serine/Threonine Kinase (MNK)-dependent pathway is also affected [35] (Fig. 2c), and to an increased phosphorylation on Ser-51 of EIF2A, an initiation factor that transfers methionyl-initiator tRNA (Met) to the small ribosomal subunit. When phosphorylated (Fig. 2c), EIF2A loses its ability to exchange GDP and GTP, impairing the formation of a complex with the EIF2B subunit and thus preventing translation initiation [36]. Analogously, SUP-T1 and Molt-4 Clone 8 T-ALL cell lines presented the same FK866-induced inhibition of the activation of 4EBP1, supporting the existence of a general mechanism underlying the FK866-induced translational arrest in leukemia cells (Fig. 2d, e). These effects were also observed using another inhibitor of NAMPT enzymatic activity, CHS-828 (Additional file 3A), but not with other commonly used chemotherapeutics as cisplatin, doxorubicin, dexamethasone and rapamycin, at equivalent pre-toxic doses. Indeed, FK866 induced a stronger protein synthesis arrest than the MTOR inhibitor rapamycin suggesting that this event is a molecular hallmark of FK866. Additionally, FK886 concomitantly induced EIF2A phosphorylation and 4EBP1 de-phosphorylation, uniquely among all the other drugs, thus mechanistically supporting the strong protein synthesis arrest. The other drugs tested were ineffective (Additional file 3B, C, D). In conclusion, these experiments show the modulation of several hubs of the signaling apparatus controlling translation initiation in response to FK866, providing a robust explanation for the marked protein synthesis inhibition observed after drug treatment.

### FK866 induces AMPK and EIF2A phosphorylation in Jurkat and primary leukemia cells

**Fig. 3:**
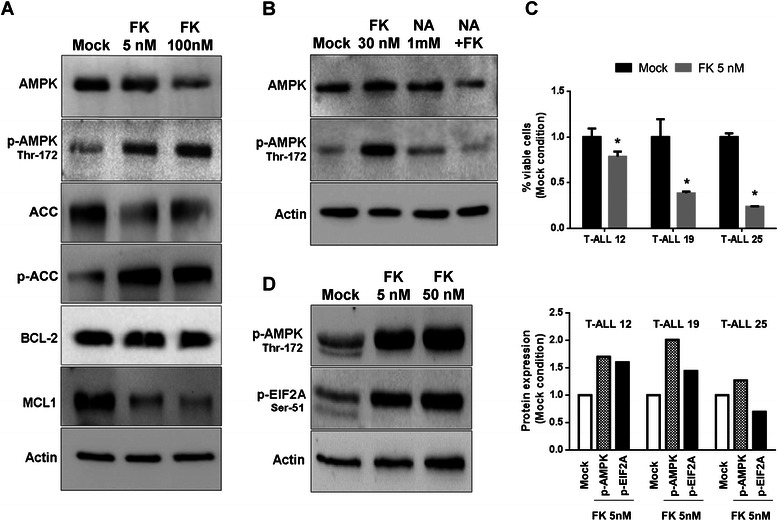
FK866 induces AMPK and EIF2A phosphorylation in primary leukemia cells. **a** Jurkat cells were treated for 48 h with or without (Mock) the indicated concentration of FK866. Thereafter, cells were lysed and the levels of total and p-AMPK (Thr-172), ACC and p-ACC, BCL-2, MCL1 and β-actin as loading control were detected by immunoblotting. One representative experiment out of three biological replicates. **b** primary B-CLL cells (source: peripheral blood; RAI stage III, 86 years, CD38-pos) were treated for 48 h with or without FK866 in the presence or absence of 1 mM NA. Thereafter, protein lysates were immunoblotted for AMPK and p-AMPK. **c** Cell viability with respect to Mock condition, measured by CellTiter Glo, of three different T-ALL xenografts (PD T-ALL 12, 19 and 25) after treatment with FK866 5nM for 48 h. **d** WB of PD T-ALL 12 as representative of T-ALL xenografts samples. Cells were treated with 5 and 50 nM FK866 for 48 h. Histogram shows the densitometric analysis of p-AMPK and p-EIF2A in the three T-ALL xenografts (PD T-ALL 12, 19 and 25)

In view of the strong translation inhibition and considering its energy-sensing activity in controlling translation [37], we investigated in Jurkat cells the impact of FK866 and CHS-828 on the phosphorylation status of AMPK, whose activation has been previously shown to be induced by FK866 in prostate and hepatic cancer cells [23, 24]. FK866 caused a partial reduction in total AMPK levels at the highest dose used, but, at a same time, a parallel dose-dependent increase of the phosphorylation of its Thr-172 and of its *bona fide* target ACC (Acetyl-CoA Carboxylase) (Fig. 3a), indicating a significant activation of AMPK. We evaluated the effect of FK866 on two important antiapoptotic factors, MCL1 (Myeloid Cell Leukemia 1) and BCL-2 (B-Cell Lymphoma 2). BCL-2 protein levels were essentially not affected by FK866 treatment as compared to the strong down-regulation of MCL1 (Fig. 3a). Notably, nicotinic acid (NA) supplementation, which blocks FK866 cytotoxic activity by allowing NAD^+^ biosynthesis through an alternative pathway (*via* nicotinic acid phosphoribosyltransferase, NAPRT1), completely prevented AMPK phosphorylation in primary B-CLL (Fig. 3b, Additional file 3E), confirming that NAD^+^ depletion is responsible for AMPK activation. In patient-derived T-ALL xenografts (PD T-ALL) the drug induced cell death and activated AMPK as well as EIF2A phosphorylation (Fig. 3c and d), demonstrating that this molecular event is not limited to cell line models but is also present in primary leukemia cells.

### EIF2A phosphorylation precedes 4EBP1 de-phosphorylation in Jurkat cells

**Fig. 4:**
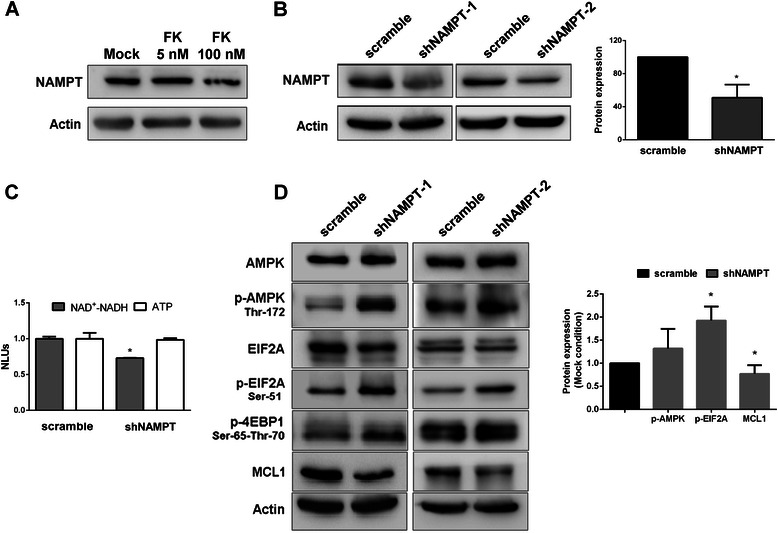
NAMPT genetic ablation induces EIF2A phosphorylation and MCL1 down-regulation. **a** Expression levels of NAMPT. Jurkat cells were treated with 5 and 100 nM FK866 for 48 h. One representative experiment out of three biological replicates is presented. **b** WB analysis indicated 50 % of NAMPT silencing (*p* < 0.001) in Jurkat cells by using lentiviral particles expressing two NAMPT-silencing shRNAs (shNAMPT-1 and −2). **c** Intracellular NAD^+^(H) and ATP levels in shNAMPT cells (transduced with shNAMPT-1 and −2) were evaluated in comparison with scramble Jurkat cells. Thirty percent reduction of NAD^+^(H) levels was observed in shNAMPT cells (*p*-value < 0.01). RLUs were normalized to number of viable cells. Mean and SD of a biological triplicate. **d** WB analysis of AMPK, p-AMPK, EIF2A, p-EIF2A, p-4EBP1 and MCL1 in shNAMPT (transduced with shNAMPT-1 and −2) cells. Histogram shows the densitometric analysis of p-AMPK, p-EIF2A and MCL1 (* indicates *p*-value <0.05). Mean and SD of a biological triplicate

NAMPT expression level during FK866 treatment remained unchanged as expected (Fig. 4a). Genetic ablation of NAMPT by lentiviral transduction in Jurkat cells (Fig. 4b) lowered NAD^+^(H) level to 75 % of the control while ATP level was not significantly decreased thus inducing an intermediate condition of energetic stress compared to the one obtained with 5 nM FK866 administration (Fig. 4c). In these conditions of mild stress, AMPK was marginally activated but, nevertheless, we observed a significant phosphorylation of EIF2A but not the de-phosphorylation of 4EBP1, suggesting that the first event precedes the second one (Fig. 4d). Importantly, we observed a clear down-regulation of MCL1, as observed with FK866 treatment (Fig. 3a), suggesting that EIF2A activation is an early response to NAD^+^(H) shortage.

### FK866-induced AMPK activation regulates EIF2A phosphorylation

**Fig. 5:**
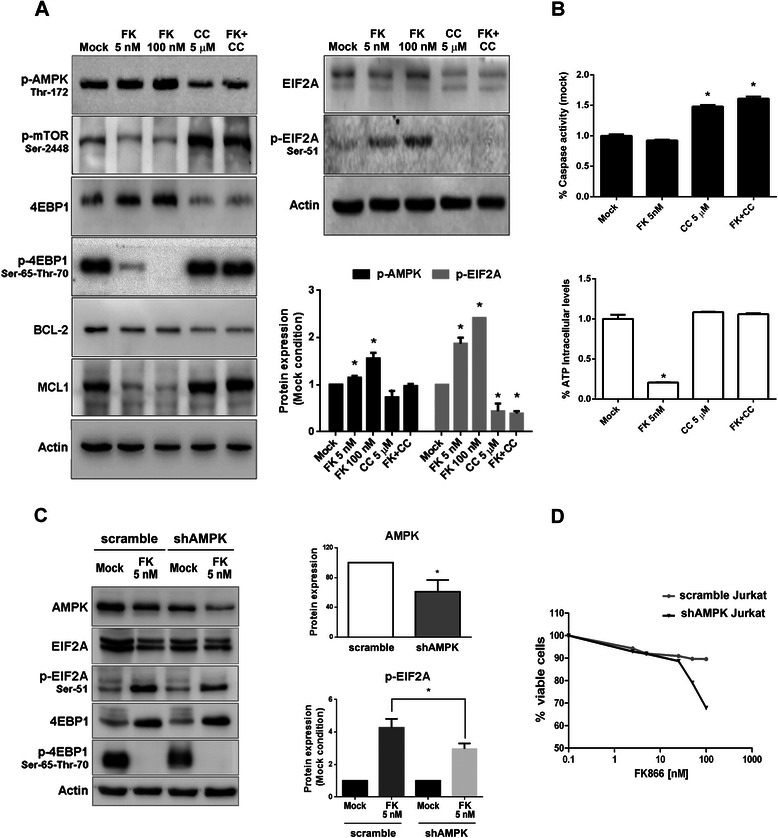
AMPK regulates EIF2A phosphorylation and is a pro-survival factor in Jurkat cells. **a** Jurkat cells were treated with or without FK866 at the indicated concentrations in the presence or absence of Compound C 5 μM for 48 h. Thereafter, cells were lysed and the levels of p-AMPK (Thr-172), p-MTOR, 4EBP1, p-4EBP1, BCL-2, MCL1, EIF2A and p-EIF2A were revealed by immunoblotting. Histogram shows the densitometric analysis of p-AMPK and p-EIF2A. **b** In the same samples, caspase 3/7 activity was quantified and relative ATP levels were determined and then normalized to the number of viable cells (* indicates *p*-value <0.05). **a** and **b** are representative of a biological triplicate (mean and SD). **c** Jurkat cells were transduced with lentiviral particles containing scramble or shAMPK (targeting the α1 and the α2 subunit), then treated for 48 h with or without 5 nM FK866. Cell lysates were used for total AMPK, EIF2A, p-EIF2A, 4EBP1, p-4EBP1 and β-actin immunoblotting. WB analysis indicated 40 % of AMPK silencing (*p*-value < 0.05) and densitometric analysis shows the significant decrease of p-EIF2A in shAMPK Jurkat cells (*p*-value < 0.03). Mean and SD of a biological triplicate. **d** Jurkat scramble and shAMPK cells were treated for 48 h with the indicated doses of FK866. Cell viability was determined by PI staining and flow-cytometry (two biological replicates and statistics based on the acquisition of 10000 events/sample)

To formally assess the role of AMPK in FK866-induced translational arrest, we pharmacologically blocked AMPK with Compound C, a small molecule inhibitor of this enzyme, although not selective [38]. In addition, we down-regulated AMPK using lentiviral transduction of shRNAs. Compound C administration to Jurkat cells treated with FK866 abrogated AMPK phosphorylation, reactivated the MTOR/4EBP1 pathway and restored EIF2A in its un-phosphorylated state (Fig. 5a). Rescue experiments with Compound C did not show any down-regulation of MCL1 protein level with no change in BCL2 expression (Fig. 5a). Co-treatment with Compound C partially reverted FK866-induced ATP loss but activated the apoptotic response (Fig. 5b). Down-regulation of AMPK was achieved by targeting both AMPKα1 and AMPKα2 isoforms (shAMPK cells). We then exposed silenced and control (scramble) cells to 5 nM of FK866 for 48 h (Fig. 5c). In shAMPK cells we observed a significant decrease of EIF2A phosphorylation but not of 4EBP1 de-phosphorylation. This supports the notion that FK866-induced AMPK activation is primarily involved in the regulation of EIF2A phosphorylation and subsequently in 4EBP1 de-phosphorylation (Fig. 5c). Importantly, shAMPK Jurkat cells showed an increased sensitivity to FK866 with respect to control cells, as revealed by PI staining and flow-cytometry (Fig. 5d), pointing out the protective effect of AMPK in FK866-induced stress conditions.

### EIF2A mediates the AMPK pro-survival effect during FK866 treatment

**Fig. 6:**
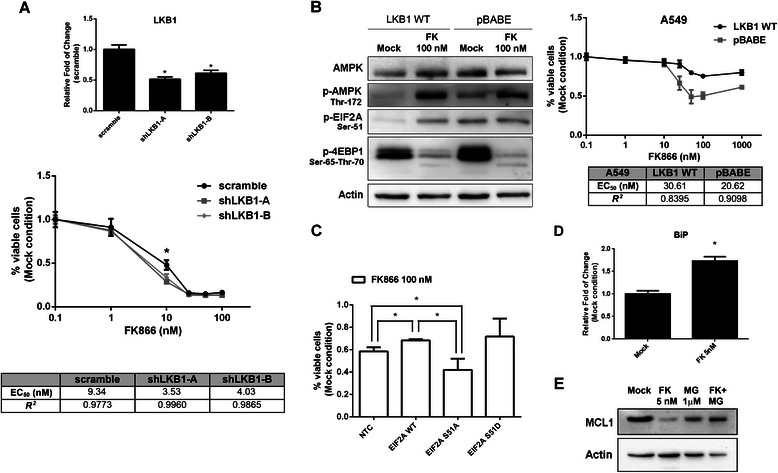
Protective role of EIF2A and FK866 induced UPR. **a** Expression level of LKB1 mRNA, evaluated in Jurkat cells after 120 h of lentiviral transduction with shRNAs expressing the control sequence (scramble) or two LKB1-silencing shRNAs (shLKB1-**a** and –**b**), in the upper panel. Cells were transduced for 72 h and then treated with FK866 for 48 h. Viability was measured by MTT assay in comparison with Mock (DMSO) condition, in the lower panel. Mean and SD of a biological triplicate (*, *p*-value < 0.05). **b** WB analysis indicated the levels of AMPK, p-AMPK, p-EIF2A, p-4EBP1 in A594 cells expressing LKB1 (LKB1 WT) or transduced with an empty vector (pBABE) treated or not (Mock) with 100 nM FK866 for 48 h, left panel. A549 cells were treated with indicated concentration of FK866 for 48 h and cell viability as shown in dose–response curve was evaluated by MTT assay, right panel. Mean and SD of three biological replicates. **c** A549 viability after 48 h of treatment with FK866 100 nM in un-transfected (NTC) cells and transfected with EIF2A wild type, EIF2A-S51A, EIF2A-S51D (mean and SD of three experiments,*, *p*-value < 0.05). **d** Expression level of BiP mRNA, evaluated in Jurkat cells after 48 h of treatment with FK866 5nM (*, *p*-value < 0.0005). Mean and SD of a biological triplicate. **e** WB analysis of MCL1 in Jurkat cells treated with FK866 for 48 h and with the proteasome inhibitor MG132 1 μM for 24 h. MG132 was added after 24 h of FK866 treatment. One representative experiment out of three biological replicates

Given the protective role of AMPK in a context of FK866-sensitive cancer cell, we hypothesized that the liver kinase B1 (LKB1), a well-established AMPK regulator, can also exert the same protective effect. Indeed, genetic ablation of LKB1 in Jurkat cells led to an increase toxicity of FK866 treatment (Fig. 6a). Accordingly, we used two lung adenocarcinoma cell lines (H460 and A549), bearing genetic inactivation of LKB1 to prove the dependency of FK866 efficacy on the activation of the LKB1/AMPK pathway. These results provide a rationale for the utilization of NAMPT inhibitors in cancers with this type of genetic background. The cells were stably transduced with retroviral vectors encoding parental LKB1 cDNA (LKB1 WT) or with a control vector (pBABE). FK866 treatment induced AMPK and EIF2A phosphorylation in addition to 4EBP1 de-phosphorylation only when LKB1 was active. On the other hand, albeit to a different extent among the two cell lines, FK866 was not able to activate AMPK or EIF2A but was still effective in de-phosphorylating 4EBP1 when LKB1 was inactive. Viability assays indicated an increased sensitivity of LKB1 negative, EIF2A un-phosphorylated cells to FK866 compared to LKB1 expressing cells (Fig. 6b and Additional file 4A). Finally, in order to assess the relevance of EIF2A in mediating the AMPK induced protection from FK866, we treated with the drug A549 and Jurkat cells transfected with EIF2A, its phosphomimetic mutant S51D or with the non-phosphorylatable mutant S51A. In the context of inactive LKB1, the overexpression of EIF2A and the EIF2A-S51D isoform led to a protective effect, while the alanine mutant induced an increase of cell toxicity (Fig. 6c). The same trend was observed also in Jurkat cells (Additional file 4B). Therefore, these data indicate that the translation arrest induced by EIF2A mediates the protective effect of AMPK from FK866 induced stress.

### EIF2A balances pro-survival and pro-death pathways

EIF2A is a key factor regulating the translation machinery in response to a myriad of factors including nutrient depletion, presence of exogenous mRNA and the Unfolded Protein Response (UPR) following the induction of Endoplasmic Reticulum (ER) stress [39]. Indeed, FK866 induced the overexpression of BiP/Grp78 mRNA, coding for a chaperone involved in the folding of ER proteins (Fig. 6d), thus indicating UPR activation in Jurkat cells after 48 h treatment. Moreover, FK866 induced MCL1 down-regulation was dependent on proteasome activation as demonstrated by the rescue of its expression level by MG132 treatment in Jurkat cells (Fig. 6e) [40]. In conclusion, FK866 induces an AMPK-EIF2A mediated translational arrest, which is responsible for MCL1 down-regulation and the activation of the UPR response, that is a strategic pausing step necessary to protect cells from FK866-induced energetic stress.

## Discussion

We investigated the link between NAD^+^(H) depletion and cell death using a T-ALL cell model after induction of the primary effects of NAMPT inhibition, namely NAD^+^(H) and ATP depletion, while nearly preserving total cell viability. The functional consequences of NAD^+^(H) depletion upon FK866 treatment resulted in a marked inhibition of the three major pathways regulating the translation process and in a striking arrest of protein synthesis. Interestingly, FK866 efficacy in blocking protein synthesis was higher than all the other chemotherapeutics tested and even higher than the MTOR inhibitor rapamycin, suggesting that this is a crucial event in the cell response to FK866. This phenomenon is general because it was observed in primary leukemic samples, coming both from B-CLL and T-ALL patients, and in T-ALL derived cancer cell lines. Nicotinic acid rescue experiments, treatment with the FK866 analog CHS-828 and NAMPT genetic ablation showed that translation arrest was dependent on the shortage of ATP and NAD^+^(H) induced by the inhibition of the NAMPT catalytic function and not by unspecific FK866 effects. FK866 has been shown to have contrasting effects on AMPK. In neuronal cells FK866 decreased AMPK activation and was detrimental for neuronal survival [25], however in cancer cells that have a dysregulated metabolic demand, it has been observed the opposite. In prostate cancer cells, FK866 treatment reduced fatty acid and phospholipid synthesis, partly *via* AMPK activation [23]. FK866 induced activation of AMPK and subsequent decreased phosphorylation of 4EBP1 by MTOR has been observed in hepatocarcinoma cells. Given the importance of MTOR in sustaining cancer cell growth, this event was proposed as an effective mechanism to target cancer cells [23]. By evaluating the early molecular effects of FK866 treatment on protein synthesis, we observed the involvement of the same pathway but, in addition, we showed a protective role for AMPK and EIF2A. In our experimental conditions, the inactivation of MTOR by AMPK and consequent protein synthesis arrest had a protective effect conferring temporary resistance to the FK866-induced energetic stress. Additionally, we determined that AMPK-induced hyper-phosphorylation of EIF2A is regulated by the fluctuations of NAD availability at the intracellular level. This molecular mechanism, leading to inhibition of translation initiation, followed AMPK activation. In fact, genetic AMPK down-regulation of both isoforms of the α catalytic subunit rescued the FK866 induced hyper-phosphorylation of EIF2A. As a further confirmation, the same results were obtained by AMPK functional ablation using the inhibitor Compound C or by inactivation of its upstream regulator LKB1. Indeed, rescue of 4EBP1 phosphorylation levels was observed only after Compound C administration, suggesting that EIF2A is a preferential target of the AMPK signaling cascade, at least in the initial phase of cell response to FK866. Interestingly, in our cell model and at the doses we used, we did not observe Compound C-induced phosphorylation of EIF2A as recently reported in different cancer cells [41]. Our cell model resembles the AICAR-induced AMPK activation that leads to EIF2A phosphorylation in adipocytes, an event shown to be crucial for AMPK-induced apoptosis [42], and supports the idea that FK866 induced activation of the AMPK-EIF2A axis can be a novel pathway to be investigated to elucidate the pharmacology of FK866.

Many types of cancer, as sporadic lung, cervical, and endometrial cancers, carry LKB1 deficiency that can be exploited with metabolic drugs since these cells are unable to appropriately respond to metabolic stress [43]. Given the protective role of LKB1/AMPK pathway against FK866, our study suggests the utilization of FK866 as a metabolism-based cancer therapeutic to selectively target LKB1-deficient tumors. Indeed in cells lacking a functional LKB1 pathway, metabolic stress has been demonstrated to result in rapid apoptosis as the cells are unable to sense energetic stresses and activate mechanisms to restore energy homeostasis [44].

Previous studies have shown that inhibition of the MTOR/4EBP1 pathway in leukemia cells leads to a reduction in the levels of the anti-apoptotic protein MCL1, with important implications for chemosensitivity [45]. Down-regulation of MCL1 through inhibition of translation has been clearly associated with enhanced lethality in Jurkat cells [46]. Importantly, FK866 administration led to smooth death [40] *via* EIF2A-dependent MCL1 down-regulation consequent to translation arrest and simultaneous proteasome activation. Indeed, MCL1 intracellular levels were shown to be strictly dependent on the activation of EIF2A [47] and AMPK [48], and to the subsequent translation arrest. This could provide a molecular explanation for the anti-leukemic activity of NAMPT inhibitors. Notably, the ectopic expression of the non-phosphorylatable mutant EIF2A-S51A increased FK866 toxicity. Therefore the activation of the AMPK-EIF2A axis is essential for the tumor cell to adapt to the shortage of NAD^+^(H). For example, the increased expression level of BiP mRNA is a specific adaptive response observed in the integrated stress response (ISR) and translational repression [49]. The exacerbation of proteasome inhibition with bortezomib has been shown to potentiate FK866 efficacy through the activation of the caspases’ cascade [40]. Here we show the relevance of EIF2A activation in this mechanism. Additionally, the synergistic effect of FK866 with cyclosporine in leukemia cells has been ascribed to the activation of the UPR [50]. This suggests that the exacerbation of the UPR, which is dependent on EIF2A, can be thought as a relevant strategy to potentiate the effect of FK866 in conditions in which activation of the EIF2A-dependent UPR is desirable, i.e., diabetes, atherosclerosis, or neurodegenerative disorders [51]. Indeed, FK866 effects on translation resemble the ones induced by metformin, a well-known AMPK activator with antidiabetic and antitumoral properties [52, 53]. Finally, de-phosphorylation of EIF4E, never linked to NAMPT inhibitors or AMPK activation before, completes the general picture of a global inhibition of the translation process, even though the mechanism leading to upstream MNK activation has not been investigated yet.

## Conclusions

In conclusion, this work describes the activation of a complex signaling network in which the AMPK-EIF2A axis is responsible for the early cellular response to the metabolic stress produced by FK866. In an experimental condition in which catastrophic proteolytic cascades are not yet started but the energetic demand is high, EIF2A acts as an early master regulator of cell fate, blocking anabolic processes and, at the same time, modulating cell death and adaptive pathways. Therefore EIF2A-dependent processes, such as protein synthesis and UPR, acquire fundamental relevance in explaining the mechanism of action of NAMPT inhibitors.

## Additional files

Additional file 1: **Cell-cycle analysis and*****Click-iT*****detection of RNA and Protein synthesis.** A) Cell-cycle analysis with PI staining of the nuclei after 48 h of treatment. Overnight serum starvation is shown as a positive control of induced cell cycle synchronization in G0/G1 phase. Cell phase analysis was done with ModFit LT 3.2 software by using the Sync Wizard model (30000 cells/sample in biological duplicate). B) Jurkat cells were treated for 48 h with or without (Mock) the indicated concentration of FK866 or for 3 h with 5 μM Actinomycin D, an RNA synthesis blocking agent, then subjected to *Click-it* biochemistry and flow-cytometry analyses including 7-AAD to identify living cells. C) Jurkat cells were treated for 48 h with or without (Mock) the indicated concentration of FK866 or for 3 h with 350 μM Cycloheximide, as a positive control for protein synthesis inhibition, then stained as in B. In B and C. Experiments were carried out on two biological replicates (50000 events/sample). (PDF 1562 kb)
Additional file 2: **Luciferase assays.** A) Light units, normalized to protein concentration, of RLuc-cMyc 5′UTR IRES-FLuc reporter vector transduced in Jurkat cells with lentiviral particles after 48 h of treatment with or without (Mock) the indicated concentration of FK866. Two hour treatment with 250 nM of Torin 1 served as a positive control for IRES-dependent protein translation (*p*-value <0.05). B) Light units, normalized to protein concentration, of FLuc-HCV-RLuc and FLuc-CrPV-RLuc reporter vectors transduced in Jurkat cells with lentiviral particles. Cap-dependent translation (FLuc) was strongly reduced with 5 nM and 100 nM FK866 (48 h) in comparison to Mock condition (*p*-value <0.0001). RLuc signal is not shown because of its low level and its variability between technical and biological replicates. Cells transduced with the pHR-SIN-F-HCV-R were serum starved for 5 h as a positive control of IRES activation, as shown in the graph (*p*-value <0.05). In A and B data are represented as mean and SD of three independent experiments. (PDF 584 kb)
Additional file 3: **Effects of CHS-828 and chemotherapeutics on protein translation.** A) Jurkat cells were treated for 48 h with or without (Mock) the indicated concentration of CHS-828. Caspase 3/7 activity was quantified (using 5 μM of Camptothecin for 4 h as a positive control of apoptosis) and relative ATP levels were determined and then normalized to the number of viable cells. The levels of total AMPK, p-AMPK, total EIF2A and p-EIF2A, total 4EBP1, p-4EBP1 were evaluated by WB. Histogram shows the densitometric analysis of p-AMPK and p-EIF2A (* indicates *p*-value <0.05). Mean and SD of a biological triplicate. B) Jurkat cells were treated with the indicated concentration of drugs for 48 h and cell viability was measured by Cell Titer Glo. Data are represented as mean and SD of three independent experiments. C) *Click-it* chemistry based on the incorporation of an aminoacid analog (AHA) was used to monitor protein synthesis. Jurkat cells were treated for 48 h with or without (Mock) the indicated concentration of FK866, Rapamycin (RAPA), Doxorubicin (DOXO), Cisplatin (CIS) and Dexamethasone (DEXA). The histogram quantifies the % of AHA positive cells (active protein-synthesizing cells) in the viable cell population. Flow-cytometry experiments were carried out on two biological replicates and statistics were based on acquisition of 20000 events/sample. D) Jurkat cells were treated as in C and the level of p-EIF2A and p-4EBP1 was evaluated. Histogram shows the densitometric analysis of p-EIF2A (* indicates *p*-value <0.05). Mean and SD of a biological triplicate. E) Primary B-CLL cells were treated for 48 h with or without 30 nM FK866 in the presence or absence of 1 mM NA. Histogram shows the densitometric analysis of p-AMPK/AMPK. (PDF 691 kb)
Additional file 4: **Protective role of EIF2A.** A) WB analysis indicated the levels of AMPK, p-AMPK, p-EIF2A, p-4EBP1 in H460 cells expressing LKB1 (LKB1 WT) or transduced with an empty vector (pBABE) treated or not (Mock) with 100 nM FK866 for 48 h, left panel. H460 cells were treated with indicated concentration of FK866 for 48 h and cell viability as shown in dose–response curve was evaluated by MTT assay, right panel. Mean and SD of three biological replicates. B) Jurkat viability after 48 h of treatment with FK866 5nM in un-transfected (NTC) cells and transfected with EIF2A wild type, EIF2A-S51A, EIF2A-S51D (mean and SD of three experiments,§, *p*-value < 0.1). (PDF 274 kb)

## Abbreviations

4EBP1: Eukaryotic translation initiation factor 4E-binding protein 1
ACC: Acetyl-CoA carboxylase
AKT: v-akt murine thymoma viral oncogene homolog 1
AMPK: AMP-activated protein kinase
BCL-2: B-Cell lymphoma 2
B-CLL: B-cell chronic lymphocytic Leukemia
BiP: Glucose-regulated protein 78 kDa
EIF2A: Eukaryotic translation initiation factor 2A
EIF4E: Eukaryotic translation initiation factor 4E
EMT: Epithelial-mesenchymal transition
ERK: Mitogen-activated protein kinase
LKB1: Liver kinase B1
MCL1: Myeloid cell leukemia 1
MNK: MAP Kinase Interacting Serine/Threonine Kinase
MTOR: Mammalian target of rapamycin
NA: Nicotinic acid
NAD: Nicotinamide adenine dinucleotide
NAMPT: Nicotinamide phosphoribosyltransferase
T-ALL: T-cell acute lymphoblastic Leukemia
UPR: Unfolded protein response

## References

[1] Kroemer G, Pouyssegur J (2008). Tumor cell metabolism: cancer’s Achilles’ heel. Cancer Cell.

[2] Tan B, Young DA, Lu Z-H, Wang T, Meier TI, Shepard RL (2013). Pharmacological inhibition of nicotinamide phosphoribosyltransferase (NAMPT), an enzyme essential for NAD+ biosynthesis, in human cancer cells: metabolic basis and potential clinical implications. J Biol Chem.

[3] Revollo JR, Grimm AA, Imai S (2004). The NAD biosynthesis pathway mediated by nicotinamide phosphoribosyltransferase regulates Sir2 activity in mammalian cells. J Biol Chem.

[4] Skokowa J, Lan D, Thakur BK, Wang F, Gupta K, Cario G (2009). NAMPT is essential for the G-CSF-induced myeloid differentiation via a NAD(+)-sirtuin-1-dependent pathway. Nat Med.

[5] Xiao Y, Elkins K, Durieux JK, Lee L, Oeh J, Yang LX (2013). Dependence of Tumor Cell Lines and Patient-Derived Tumors on the NAD Salvage Pathway Renders Them Sensitive to NAMPT Inhibition with GNE-618. Neoplasia.

[6] Soncini D, Caffa I, Zoppoli G, Cea M, Cagnetta A, Passalacqua M (2014). Nicotinamide phosphoribosyltransferase promotes epithelial-to-mesenchymal transition as a soluble factor independent of its enzymatic activity. J Biol Chem.

[7] Wang B, Hasan MK, Alvarado E, Yuan H, Wu H, Chen WY (2011). NAMPT overexpression in prostate cancer and its contribution to tumor cell survival and stress response. Oncogene.

[8] Nakajima TE, Yamada Y, Hamano T, Furuta K, Gotoda T, Katai H (2009). Adipocytokine levels in gastric cancer patients: resistin and visfatin as biomarkers of gastric cancer. J Gastroenterol.

[9] Montecucco F, Cea M, Cagnetta A, Damonte P, Nahimana A, Ballestrero A (2013). Nicotinamide phosphoribosyltransferase as a target in inflammation- related disorders. Curr Top Med Chem.

[10] Bruzzone S, Fruscione F, Morando S, Ferrando T, Poggi A, Garuti A (2009). Catastrophic NAD+ depletion in activated T lymphocytes through Nampt inhibition reduces demyelination and disability in EAE. PLoS One.

[11] Zoppoli G, Cea M, Soncini D, Fruscione F, Rudner J, Moran E (2010). Potent synergistic interaction between the Nampt inhibitor APO866 and the apoptosis activator TRAIL in human leukemia cells. Exp Hematol.

[12] Nahimana A, Attinger A, Aubry D, Greaney P, Ireson C, Thougaard AV (2009). The NAD biosynthesis inhibitor APO866 has potent antitumor activity against hematologic malignancies. Blood.

[13] Nencioni A, Cea M, Montecucco F, Longo VD, Patrone F, Carella AM (2013). Autophagy in blood cancers: biological role and therapeutic implications. Haematologica.

[14] Wosikowski K, Mattern K, Schemainda I, Hasmann M, Rattel B, Löser R (2002). WK175, a novel antitumor agent, decreases the intracellular nicotinamide adenine dinucleotide concentration and induces the apoptotic cascade in human leukemia cells. Cancer Res.

[15] Christensen MK, Erichsen KD, Olesen UH, Tjørnelund J, Fristrup P, Thougaard A (2013). Nicotinamide phosphoribosyltransferase inhibitors, design, preparation, and structure-activity relationship. J Med Chem.

[16] Montecucco F, Cea M, Bauer I, Soncini D, Caffa I, Lasigliè D (2013). Nicotinamide phosphoribosyltransferase (NAMPT) inhibitors as therapeutics: rationales, controversies, clinical experience. Curr Drug Targets.

[17] Cerna D, Li H, Flaherty S, Takebe N, Coleman CN, Yoo SS (2012). Inhibition of nicotinamide phosphoribosyltransferase (NAMPT) activity by small molecule GMX1778 regulates reactive oxygen species (ROS)-mediated cytotoxicity in a p53- and nicotinic acid phosphoribosyltransferase1 (NAPRT1)-dependent manner. J Biol Chem.

[18] Del Nagro C, Xiao Y, Rangell L, Reichelt M, O’Brien T (2014). Depletion of the Central Metabolite NAD Leads to Oncosis-mediated Cell Death. J Biol Chem.

[19] Ginet V, Puyal J, Rummel C, Aubry D, Breton C, Cloux A-J (2014). A critical role of autophagy in antileukemia/lymphoma effects of APO866, an inhibitor of NAD biosynthesis. Autophagy.

[20] Settembre C, De Cegli R, Mansueto G, Saha PK, Vetrini F, Visvikis O (2013). TFEB controls cellular lipid metabolism through a starvation-induced autoregulatory loop. Nat Cell Biol.

[21] Cea M, Cagnetta A, Fulciniti M, Tai Y-T, Hideshima T, Chauhan D (2012). Targeting NAD+ salvage pathway induces autophagy in multiple myeloma cells via mTORC1 and extracellular signal-regulated kinase (ERK1/2) inhibition. Blood.

[22] Hardie DG (2004). The AMP-activated protein kinase pathway--new players upstream and downstream. J Cell Sci.

[23] Bowlby SC, Thomas MJ, D’Agostino RB, Kridel SJ (2012). Nicotinamide phosphoribosyl transferase (Nampt) is required for de novo lipogenesis in tumor cells. PLoS One.

[24] Schuster S, Penke M, Gorski T, Gebhardt R, Weiss TS, Kiess W (2015). FK866-induced NAMPT inhibition activates AMPK and downregulates mTOR signaling in hepatocarcinoma cells. Biochem Biophys Res Commun.

[25] Wang P, Xu T-Y, Guan Y-F, Tian W-W, Viollet B, Rui Y-C (2011). Nicotinamide phosphoribosyltransferase protects against ischemic stroke through SIRT1-dependent adenosine monophosphate-activated kinase pathway. Ann Neurol.

[26] Spriggs KA, Stoneley M, Bushell M, Willis AE (2008). Re-programming of translation following cell stress allows IRES-mediated translation to predominate. Biol Cell.

[27] Agnusdei V, Minuzzo S, Frasson C, Grassi A, Axelrod F, Satyal S (2013). Therapeutic antibody targeting of Notch1 in T-acute lymphoblastic leukemia xenografts. Leukemia.

[28] D’Agostino VG, Adami V, Provenzani A (2013). A novel high throughput biochemical assay to evaluate the HuR protein-RNA complex formation. PLoS One.

[29] Zufferey R, Nagy D, Mandel RJ, Naldini L, Trono D (1997). Multiply attenuated lentiviral vector achieves efficient gene delivery in vivo. Nat Biotechnol.

[30] Sarbassov DD, Guertin DA, Ali SM, Sabatini DM (2005). Phosphorylation and regulation of Akt/PKB by the rictor-mTOR complex. Science.

[31] Moffat J, Grueneberg DA, Yang X, Kim SY, Kloepfer AM, Hinkle G (2006). A lentiviral RNAi library for human and mouse genes applied to an arrayed viral high-content screen. Cell.

[32] Sonenberg N, Hinnebusch AG (2009). Regulation of translation initiation in eukaryotes: mechanisms and biological targets. Cell. Elsevier Inc.

[33] Cea M, Cagnetta A, Patrone F, Nencioni A, Gobbi M, Anderson KC (2013). Intracellular NAD(+) depletion induces autophagic death in multiple myeloma cells. Autophagy.

[34] Shi Y, Yan H, Frost P, Gera J, Lichtenstein A (2005). Mammalian target of rapamycin inhibitors activate the AKT kinase in multiple myeloma cells by up-regulating the insulin-like growth factor receptor/insulin receptor substrate-1/phosphatidylinositol 3-kinase cascade. Mol Cancer Ther.

[35] Waskiewicz AJ, Flynn A, Proud CG, Cooper JA (1997). Mitogen-activated protein kinases activate the serine/threonine kinases Mnk1 and Mnk2. EMBO J.

[36] Krishnamoorthy T, Pavitt GD, Zhang F, Dever TE, Hinnebusch AG (2001). Tight binding of the phosphorylated alpha subunit of initiation factor 2 (eIF2alpha) to the regulatory subunits of guanine nucleotide exchange factor eIF2B is required for inhibition of translation initiation. Mol Cell Biol.

[37] Zhang C-S, Jiang B, Li M, Zhu M, Peng Y, Zhang Y-L (2014). The Lysosomal v-ATPase-Ragulator Complex Is a Common Activator for AMPK and mTORC1, Acting as a Switch between Catabolism and Anabolism. Cell Metab.

[38] Liu X, Chhipa RR, Nakano I, Dasgupta B (2014). The AMPK inhibitor compound C is a potent AMPK-independent antiglioma agent. Mol Cancer Ther.

[39] Wang S, Kaufman RJ (2012). The impact of the unfolded protein response on human disease. J Cell Biol.

[40] Cagnetta A, Cea M, Calimeri T, Acharya C, Fulciniti M, Tai Y-T (2013). Intracellular NAD^+^ depletion enhances bortezomib-induced anti-myeloma activity. Blood.

[41] Dai RY, Zhao XF, Li JJ, Chen R, Luo ZL, Yu LX (2013). Implication of transcriptional repression in compound C-induced apoptosis in cancer cells. Cell Death Dis..

[42] Dagon Y, Avraham Y, Berry EM (2006). AMPK activation regulates apoptosis, adipogenesis, and lipolysis by eIF2alpha in adipocytes. Biochem Biophys Res Commun.

[43] Shackelford DB, Abt E, Gerken L, Vasquez DS, Seki A, Leblanc M (2013). LKB1 inactivation dictates therapeutic response of non-small cell lung cancer to the metabolism drug phenformin. Cancer Cell.

[44] Shaw RJ, Kosmatka M, Bardeesy N, Hurley RL, Witters LA, DePinho RA (2004). The tumor suppressor LKB1 kinase directly activates AMP-activated kinase and regulates apoptosis in response to energy stress. Proc Natl Acad Sci U S A.

[45] Mills JR, Hippo Y, Robert F, Chen SMH, Malina A, Lin C-J (2008). mTORC1 promotes survival through translational control of Mcl-1. Proc Natl Acad Sci U S A.

[46] Zhou T, Li G, Cao B, Liu L, Cheng Q, Kong H (2013). Downregulation of Mcl-1 through inhibition of translation contributes to benzyl isothiocyanate-induced cell cycle arrest and apoptosis in human leukemia cells. Cell Death Dis..

[47] Fritsch RM, Schneider G, Saur D, Scheibel M, Schmid RM (2007). Translational repression of MCL-1 couples stress-induced eIF2 alpha phosphorylation to mitochondrial apoptosis initiation. J Biol Chem.

[48] Pradelli LA, Bénéteau M, Chauvin C, Jacquin MA, Marchetti S, Muñoz-Pinedo C (2010). Glycolysis inhibition sensitizes tumor cells to death receptors-induced apoptosis by AMP kinase activation leading to Mcl-1 block in translation. Oncogene.

[49] Palam LR, Baird TD, Wek RC (2011). Phosphorylation of eIF2 facilitates ribosomal bypass of an inhibitory upstream ORF to enhance CHOP translation. J Biol Chem.

[50] Cagnetta A, Caffa I, Acharya C, Soncini D, Acharya P, Adamia S (2015). APO866 Increases Antitumor Activity of Cyclosporin-A by Inducing Mitochondrial and Endoplasmic Reticulum Stress in Leukemia Cells. Clin Cancer Res.

[51] Fullwood MJ, Zhou W, Shenolikar S (2012). Targeting phosphorylation of eukaryotic initiation factor-2α to treat human disease. Prog Mol Biol Transl Sci..

[52] Dowling RJO, Zakikhani M, Fantus IG, Pollak M, Sonenberg N (2007). Metformin inhibits mammalian target of rapamycin-dependent translation initiation in breast cancer cells. Cancer Res.

[53] Larsson O, Morita M, Topisirovic I, Alain T, Blouin M-J, Pollak M (2012). Distinct perturbation of the translatome by the antidiabetic drug metformin. Proc Natl Acad Sci U S A.

