# Supplementary figures and images for: EIF2A-dependent translational arrest protects leukemia cells from the energetic stress induced by NAMPT inhibition
Source: BMC Cancer. 2015 Nov 5;15:855. doi: 10.1186/s12885-015-1845-1 (PMC4636066; doi:10.1186/s12885-015-1845-1)

Additional file 1

A

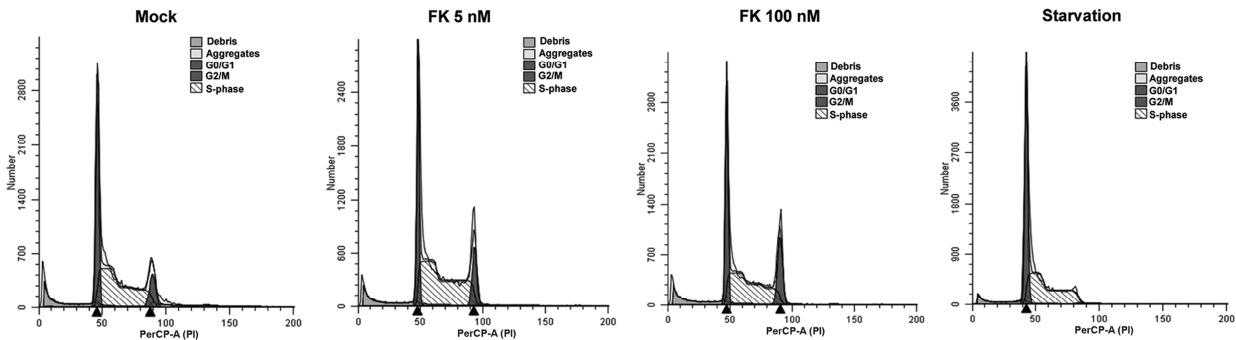

B

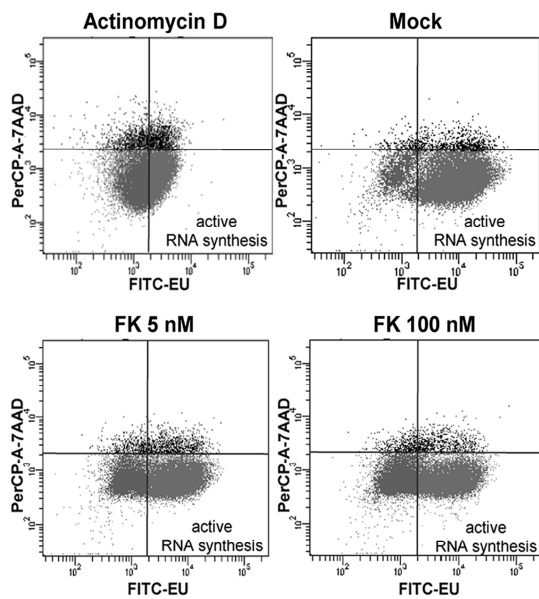

C

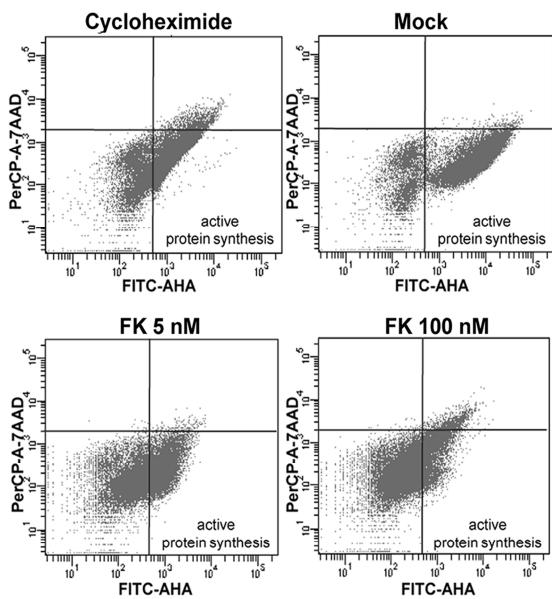

Supplement: Additional file 1: — Cell-cycle analysis andClick-iTdetection of RNA and Protein synthesis. A) Cell-cycle analysis with PI staining of the nuclei after 48 h of treatment. Overnight serum starvation is shown as a positive control of induced cell cycle synchronization in G0/G1 phase. Cell phase analysis was done with ModFit LT 3.2 software by using the Sync Wizard model (30000 cells/sample in biological duplicate). B) Jurkat cells were treated for 48 h with or without (Mock) the indicated concentration of FK866 or for 3 h with 5 μM Actinomycin D, an RNA synthesis blocking agent, then subjected to Click-it biochemistry and flow-cytometry analyses including 7-AAD to identify living cells. C) Jurkat cells were treated for 48 h with or without (Mock) the indicated concentration of FK866 or for 3 h with 350 μM Cycloheximide, as a positive control for protein synthesis inhibition, then stained as in B. In B and C. Experiments were carried out on two biological replicates (50000 events/sample). (PDF 1562 kb) [file 12885_2015_1845_MOESM1_ESM.pdf]

Additional file 2

A

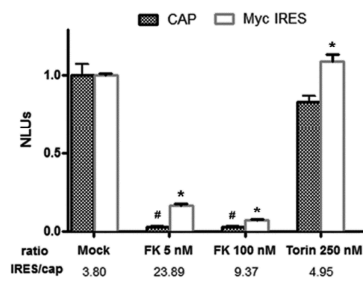

B

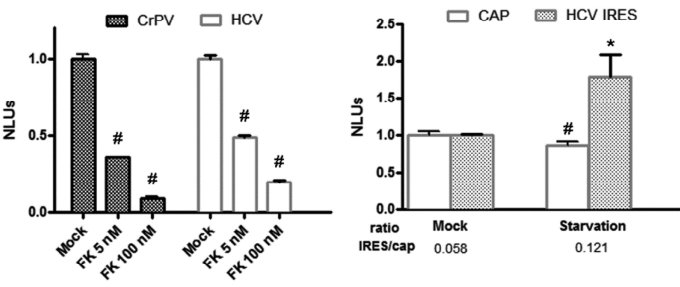

Supplement: Additional file 2: — Luciferase assays. A) Light units, normalized to protein concentration, of RLuc-cMyc 5′UTR IRES-FLuc reporter vector transduced in Jurkat cells with lentiviral particles after 48 h of treatment with or without (Mock) the indicated concentration of FK866. Two hour treatment with 250 nM of Torin 1 served as a positive control for IRES-dependent protein translation (p-value <0.05). B) Light units, normalized to protein concentration, of FLuc-HCV-RLuc and FLuc-CrPV-RLuc reporter vectors transduced in Jurkat cells with lentiviral particles. Cap-dependent translation (FLuc) was strongly reduced with 5 nM and 100 nM FK866 (48 h) in comparison to Mock condition (p-value <0.0001). RLuc signal is not shown because of its low level and its variability between technical and biological replicates. Cells transduced with the pHR-SIN-F-HCV-R were serum starved for 5 h as a positive control of IRES activation, as shown in the graph (p-value <0.05). In A and B data are represented as mean and SD of three independent experiments. (PDF 584 kb) [file 12885_2015_1845_MOESM2_ESM.pdf]

A

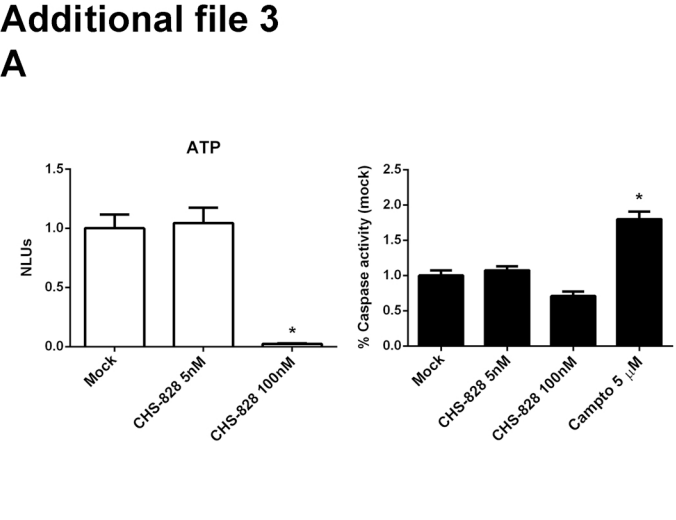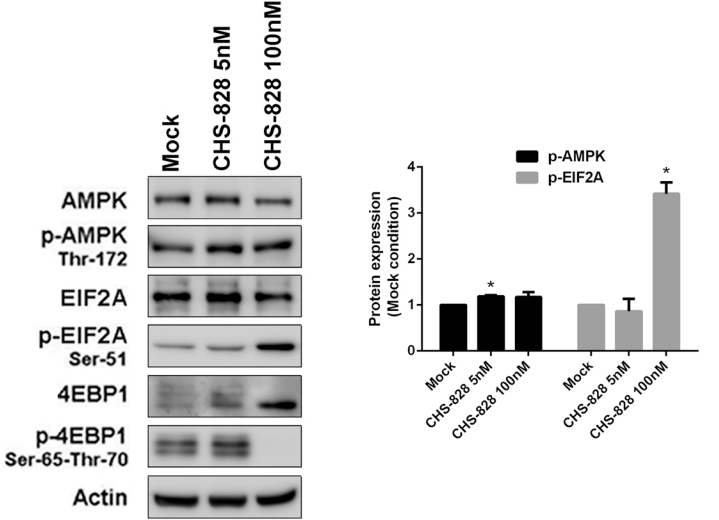

B

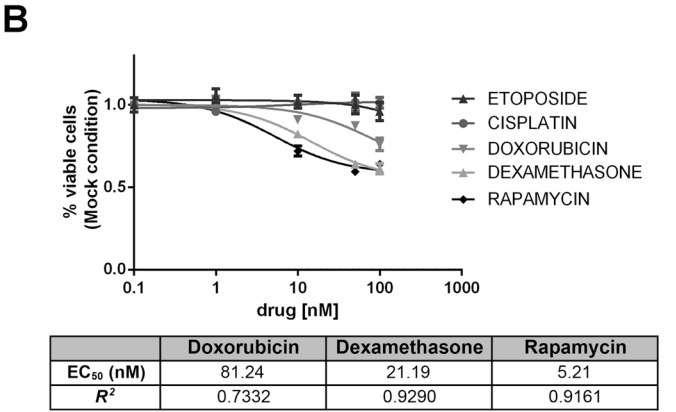

D

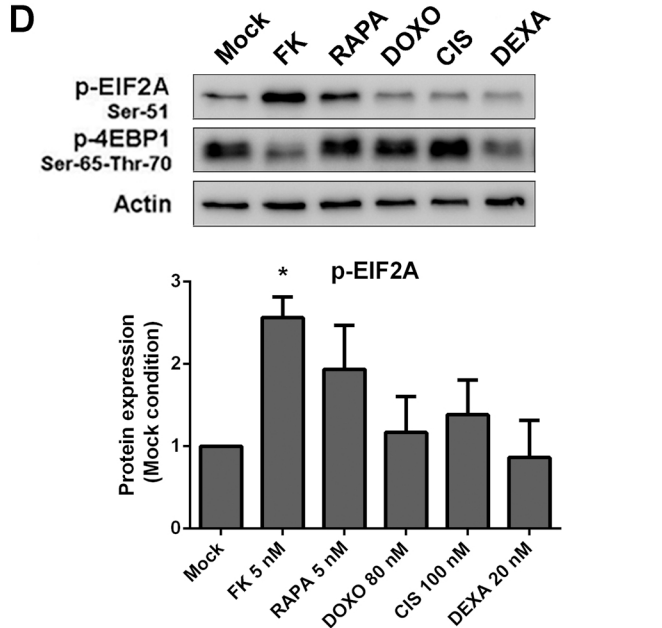

C

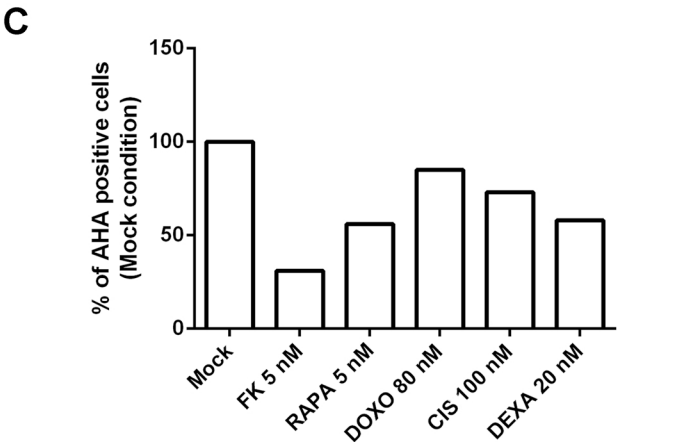

E

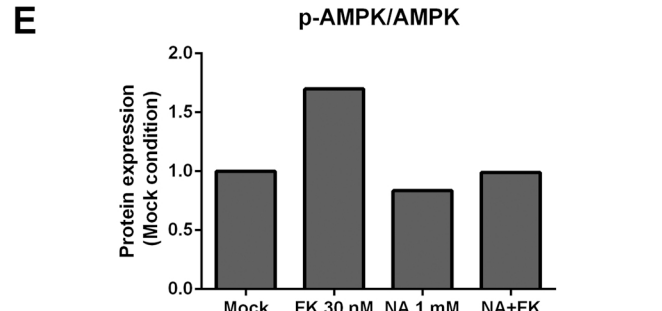

Supplement: Additional file 3: — Effects of CHS-828 and chemotherapeutics on protein translation. A) Jurkat cells were treated for 48 h with or without (Mock) the indicated concentration of CHS-828. Caspase 3/7 activity was quantified (using 5 μM of Camptothecin for 4 h as a positive control of apoptosis) and relative ATP levels were determined and then normalized to the number of viable cells. The levels of total AMPK, p-AMPK, total EIF2A and p-EIF2A, total 4EBP1, p-4EBP1 were evaluated by WB. Histogram shows the densitometric analysis of p-AMPK and p-EIF2A (* indicates p-value <0.05). Mean and SD of a biological triplicate. B) Jurkat cells were treated with the indicated concentration of drugs for 48 h and cell viability was measured by Cell Titer Glo. Data are represented as mean and SD of three independent experiments. C) Click-it chemistry based on the incorporation of an aminoacid analog (AHA) was used to monitor protein synthesis. Jurkat cells were treated for 48 h with or without (Mock) the indicated concentration of FK866, Rapamycin (RAPA), Doxorubicin (DOXO), Cisplatin (CIS) and Dexamethasone (DEXA). The histogram quantifies the % of AHA positive cells (active protein-synthesizing cells) in the viable cell population. Flow-cytometry experiments were carried out on two biological replicates and statistics were based on acquisition of 20000 events/sample. D) Jurkat cells were treated as in C and the level of p-EIF2A and p-4EBP1 was evaluated. Histogram shows the densitometric analysis of p-EIF2A (* indicates p-value <0.05). Mean and SD of a biological triplicate. E) Primary B-CLL cells were treated for 48 h with or without 30 nM FK866 in the presence or absence of 1 mM NA. Histogram shows the densitometric analysis of p-AMPK/AMPK. (PDF 691 kb) [file 12885_2015_1845_MOESM3_ESM.pdf]

Additional file 4

A

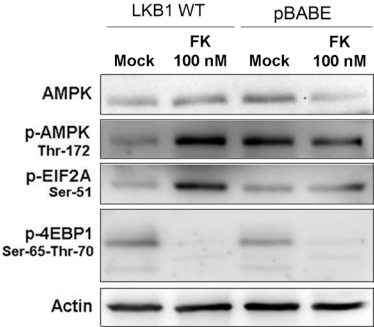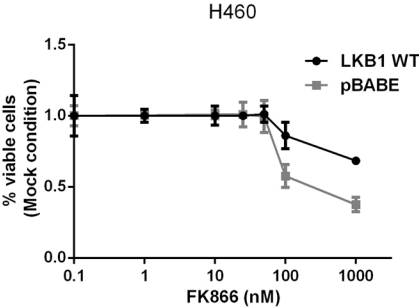

| H460                  | LKB1 WT | pBABE |
|-----------------------|---------|-------|
| EC <sub>50</sub> (nM) | 102.0   | 84.3  |
| R <sup>2</sup>        | 0.7841  | 0.945 |

B

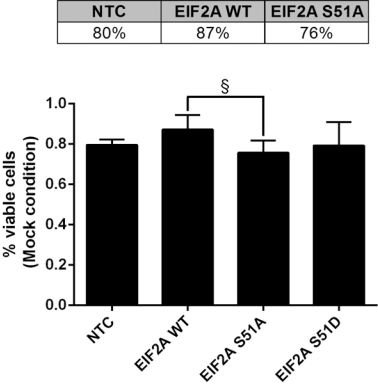

Supplement: Additional file 4: — Protective role of EIF2A. A) WB analysis indicated the levels of AMPK, p-AMPK, p-EIF2A, p-4EBP1 in H460 cells expressing LKB1 (LKB1 WT) or transduced with an empty vector (pBABE) treated or not (Mock) with 100 nM FK866 for 48 h, left panel. H460 cells were treated with indicated concentration of FK866 for 48 h and cell viability as shown in dose–response curve was evaluated by MTT assay, right panel. Mean and SD of three biological replicates. B) Jurkat viability after 48 h of treatment with FK866 5nM in un-transfected (NTC) cells and transfected with EIF2A wild type, EIF2A-S51A, EIF2A-S51D (mean and SD of three experiments,§, p-value < 0.1). (PDF 274 kb) [file 12885_2015_1845_MOESM4_ESM.pdf]
